# Reply to Del Guacchio et al. The Genus *Lophiolepis* Is at Least as Well Supported as *Afrocarduus*, *Afrocirsium*, and *Nuriaea*. Comment on “Moreyra et al. African Mountain Thistles: Three New Genera in the *Carduus*-*Cirsium* Group. *Plants* 2023, *12*, 3083”

**DOI:** 10.3390/plants13233400

**Published:** 2024-12-04

**Authors:** Lucía D. Moreyra, Alfonso Susanna

**Affiliations:** Botanic Institute of Barcelona (IBB), CSIC-Ajuntament de Barcelona, Pg. Migdia, s.n., 08038 Barcelona, Spain; luciad.moreyra@ibb.csic.es

## 1. Introduction

We recently published a paper that presented the first Hyb-Seq phylogeny for subtribe Carduinae, in which we described three new genera endemic to the African Sky-Islands [1] While this was our primary objective, we also aimed to assess the generic delimitation of the subtribe. In 2022, ref. [2] published a phylogeny in the journal of the Societa Botanica Italiana using a set of molecular markers that we [3] had demonstrated were insufficient to resolve questions regarding the origin and evolutionary history of this group. The main issue with their study was the lack of resolution, as five out of seven markers they used are plastidic, which are known to tell only one side of the evolutionary story.

In contrast, we provided two nuclear phylogenies using 350 markers and a third one using the whole chloroplast, demonstrating that *Cirsium* remains monophyletic in the nuclear dataset. We found only one incongruence regarding *Picnomon*, a monotypic genus of putative hybrid origin between *Notobasis* and *Cirsium*, reflected as an artifact in the coalescence phylogeny. These implications are currently under investigation and explaining them in detail here would be too extensive and off-topic.

The authors of the comment argue that our plastid phylogeny resembles the structure and organization of their published phylogeny, implying that we are ignoring these results and suggesting that plastid history prevails over nuclear history. We are using nuclear data to assess the complete history of the genus and chloroplast data to evaluate incongruencies. However, neither our phylogeny nor Del Guacchio et al. [2] fully explains the origin and evolution of the genus due to a lack of bioinformatic and molecular tools to elucidate how reticulation impacts our phylogenies and to account for the incongruences observed. Consequently, since both subgenera *Cirsium* and *Lophiolepis* remain sister groups in the nuclear reconstruction and given that the plastid data fail to recover natural groups, both subgenera should remain as a single entity until we have strong evidence of the contrary.

## 2. Section: “2.1 Scopes and Limits of Del Guacchio et al. [2]”

When we discussed how Del Guacchio et al. [2] provided a phylogeny that “fails to support the classification proposed since *Cirsium* s. str is not recovered as monophyletic and the entire genus *Carduus* was nested within *Cirsium* s. str”, we were illustrating that their phylogeny failed to recover natural (monophyletic) entities within the Carduus-Cirsium group. However, because the *Lophiolepis* clade was supported, they interpreted this as sufficient molecular support to segregate it as an independent genus.

The authors also state, “This is a misleading claim” because Del Guacchio et al. [2] “only proposed a generic recognition of *Lophiolepis* and *Epithrachys* and did not infer any conclusion regarding the monophyly of the remaining *Cirsium* species. This is already obvious from the title of our contribution (‘Towards a monophyletic classification of Cardueae’)”. From our point of view, using a phylogeny that only supports one clade and fails to recover the accepted groups is a misleading interpretation that conveniently supports only their hypothesis. Valuable information on the classification of Carduinae can be provided only if new evidence is added that complements the information we currently have. From the figure the authors provided in this comment paper (Figure 1) [4], it seems that they are proposing that to achieve monophyletic groups we should keep splitting *Cirsium*, which is plainly an overreach.

## 3. Section: “2.2 The Separation of Lophiolepis Is Not Based on “Weakly Supported Molecular Results””

We must strongly disagree with the title of this section. We will now discuss the points made by the authors:

**“First of all, we take issue with the claim that we split *Cirsium* “into four genera””.** Maybe we should have said three genera and one nothogenus, but this does not change the fact that we split *Cirsium* into four entities.

**“Second, five authors [**2**], which are also co-authors of [**1**], used only six markers (two nuclear and four plastid); therefore, ref. [**2**] simply could not prove the insufficiency of the seven markers we used in [**3,4**], despite what stated by Moreyra et al. [**1**]”.** As we said before, our co-authors explicitly stated, “A ‘successful solution’ for delimiting generic boundaries in the Carduus-Cirsium group is long overdue, but a stable taxonomy for the group at the generic level is only just beginning to emerge. Although we show that both *Carduus* and *Cirsium* do not form monophyletic groups, a definitive generic delimitation solution remains elusive at this time. Before adopting either of the solutions presented for delimiting generic boundaries, we suggest waiting for the results of an upcoming study in the Carduus-Cirsium group. The authors are currently working on a phylogenomic study using targeted enrichment of highly informative nuclear regions”.

Our co-authors are clearly saying that these markers are not enough. The only addition made by Del Guacchio et al. [2] was another plastid marker, which our co-authors also said was not appropriate [3], “There was no significant (BS ≥ 75 and PP ≥ 0.75) incongruence between the nuclear and plastid gene trees (Supplementary Figure S1A–F). This is either the result of a lack of parsimony-informative characters in the plastid regions or missing sequence data for the four plastid markers”. The fact that Del Guacchio et al. [2] did not find any incongruence between nuclear and plastid markers supports the idea that their dataset lacks informative data. We discuss this further in the fourth point.

**Third Point:** We agree. But again, the authors are assuming that Sanger phylogenies based on plastid markers are somehow stronger than our nuclear results using Hyb-Seq.

**Fourth Point:** The authors list the number of species included in their article [2] and the number of sequences included, saying, **“Taken together, the claim of Moreyra et al. [**1**] that our results [**3**] are “mainly based on the weakly supported molecular results from [**2**]” is, therefore, inaccurate and misleading”.** We are sorry about this, but in molecular science “weakly supported molecular results” do not imply a lack of individuals or markers but rather a lack of resolution, which is the only thing that really matters in discussing taxonomy and evolution. The markers the authors used show a phylogeny with many polytomies, and the authors only show the support of the clades in the Supplementary Material, something we found very curious, as in general, phylogenies must always show the support if the figure is displayed in the main paper.

**Fifth Point:** We discussed in Moreyra et al. [1] that the matrix of Del Guacchio et al. [2] lacks almost 50% of the sequences, leaving the missing sequences in the main matrix as missing data, and explained how they assessed this issue with molecular tools. We agree with this procedure as we always encourage people in the field to ensure the proper treatment of the data. But later in the text, the authors say, **“Moreover, despite the statements by [**1**], our topology largely concurs with their plastid phylogeny (Figure 1c,d), obviously if the *Lophiolepis* clade is labeled as such in the trees, something they decided not to do in their figures [**1**]. This similarity seems to us final in order to reject any alleged inadequateness of our results to reach the aim of our previous study, i.e., to characterize the *Lophiolepis* clade”.** Once again, the authors admit that their phylogeny only agrees with our plastid one, and therefore, they are missing half of the evolutionary history. Moreover, processes like plastid capture can distort their results and that is why evolutionary biologists use nuclear data, especially now that we have the technology to process thousands of molecular characters. It is widely known and accepted that the plastid is very conserved and is not enough to infer a holistic picture of evolution.

So, to this statement made by the authors, “In conclusion, the separation of *Lophiolepis* from *Cirsium* has been clearly supported independently by [1,2] as well as by Moreyra et al. [1] themselves (Figure 1)”, we must express our disagreement because our chloroplastic data only show a conserved chloroplast fixed in many groups, and our nuclear data show a monophyletic *Cirsium*, including the so-called *Lophiolepis* clade within it.

## 4. Section: 2.3 There Is No Evidence of an “Extremely Frequent Hybridization Between *Cirsium* and *Lophiolepis*”

In Moreyra et al. [1], we proposed that the frequent hybrids between *Cirsium* and *Lophiolepis* provide evidence of the genetic closeness between these two subgenera. To support this, we cited Del Guacchio et al. [2], who stated: **“Numerous intergeneric hybrids have been described between *Lophiolepis* and *Cirsium* (especially *C*. *vulgare*)”.** Now, the authors are retracting this statement, which we find very unprofessional, as it undermines their credibility.

Our interpretation of the matter is the following: *Cirsium vulgare* was proven to be an allopolyploid between subgenera *Cirsium* and *Lophiolepis*, and as a consequence, it shows remnants of both genomes. *Cirsium vulgare* hybridizes with species from both subgenera, still showing evidence of closeness. Of course, because these subgenera diverged around 7 million years ago, we expect that hybridization within each clade to be higher than between the two.

## 5. Section: “2.4 The Carduineae Treatment by Moreyra et al. [1] Is Inconsistent and Contradictory”

We are astonished by this title. The authors comment on their disagreement on our decision to maintain *Cirsium* as a single entity despite one of its subgenera, *Lophiolepis*, having a unique apomorphy. They contrast this with our decision to segregate African thistles into two different genera, which they argue **“can be morphologically separated chiefly (or exclusively!) on account of their phyllary appendages (absent vs. fimbriate) and, less clearly, by pappus bristles (scabrid-short barbellate vs. plumose) and stems (unwinged or interruptedly winged vs. winged)”.** The unique apomorphy of *Lophiolepis* is not exclusive to the clade, as several species of subgenus *Cirsium* also show this character, for instance, *C. interpositum*, *C. lidjiangense*, *C. subulariforme*, *C. vulgare*, and *C. italicum* [5,6]. On the contrary, we found a unique combination of five characters to describe these new genera.

To us, it is clear that *Afrocarduus* is very different from *Afrocirsium*, and even if we did not have additional evidence, which we have with the molecular results, the morphological differentiation is sufficient for us. We are working on reconstructing the biogeographical history of the Carduus-Cirsium group, which will be published this year. Our findings illustrate that *Afrocirsium* has an earlier origin than *Afrocarduus*, while both subgenera of *Cirsium* began to diversify at the same time. Even if these subgenera are on different evolutionary tracks, it may take a few more million years for them to be fully separated. Here, the authors are comparing two groups that are easily distinguishable from one another (*Afrocarduus* and *Afrocirsium*) with two very diverse and widespread subgenera that have a common ancestor, form a monophyletic group, and are almost impossible to differentiate from one another in the field.

Additionally, the authors propose that we are inconsistent in our treatment of hybrids. They note that we suggested one species of *Afrocirsium* could have a hybrid origin and yet decided to segregate it while we chose to maintain subgenus *Lophiolepis* within *Cirsium* because they frequently hybridize, indicating genetic closeness. The authors are mixing two different processes, ancient hybridization events, which are widespread and do not impact classifications; and natural, recent occurrence of hybrids. For further information, we recommend reading the relevant literature on these processes.

The authors correctly point out that our phylogeny lacks *Cirsium italicum*, which is why we cannot comment on the genus *Eriolepis* as segregated by Del Guacchio et al. [2]. We explained this in Moreyra et al. [1], so we do not see the inconsistency. We would like to give the premise that in our recent work, we did include this species, and it is not a separate evolutionary entity, as *Cirsium vulgare* is not either.

## 6. Section: “The Genus *Cirsium*, as Circumscribed by Moreyra et al. [1], Is Definitively Polyphyletic as a Consequence of Their Own Results”

We thoroughly considered this title and the only logical conclusion we found is that the authors of the comment paper have different definitions of polyphyletic than us. Before addressing this, we need to mention the following key points: (1) Plastid genomes are usually inherited uniparentally (mostly maternally), which can lead to biased phylogenetic signals. In contrast, nuclear genomes, inherited biparentally and independently from the plastid genome, offer a more balanced view of inheritance; (2) Plastid genomes are more prone to horizontal gene transfer, which complicates phylogenetic analyses, while nuclear genomes are less affected by this, leading to more reliable phylogenetic reconstructions; (3) Plastid genes typically evolve more slowly than nuclear genes. While this slower rate can be advantageous for certain deep phylogenetic analyses, it can also mean that plastid phylogenies are less sensitive to recent evolutionary events.

Our nuclear dataset resolves *Cirsium* as monophyletic while our plastid dataset shows *Cirsium* split into many clades, which does not reflect the evolutionary relationships between the genera that we can infer from the nuclear analyses. This last result only shows that a different chloroplast is fixed in different groups of *Cirsium*, something that could be the result of horizontal gene transfer in the early stages of diversification. Of course, this is a very important result, and it is relevant to explain relationships between groups, but by no means implies that they constitute different evolutionary entities. We believe that it is very easy to fall into overinterpretation of the results and holistic approaches must be taken. Moreover, we must accept that the scientific community is still lacking the tools to answer many questions. As a community, we thought that we would have answers once we had more data, but the events of the past years illustrate the need to be careful while interpreting our data. That is the main reason why we are working with more than 20 co-authors of more than 10 nationalities in an effort to close the knowledge gap and improve the quality of our research.

On a side note, we want to express our disagreement with how these authors are citing our work. The authors claim in one of their recent papers [7] that **“Recent phylogenetic investigations by Ackerfield et al. [**3**], Del Guacchio et al. [**2**], Bureš et al. [**7**], and Moreyra et al. [**1**] have revealed the polyphyletic nature of the broad taxonomic concept of the genus *Cirsium* Mill. Consequently, Del Guacchio et al. [**2**] initially proposed the separation of two smaller genera from the large genus *Cirsium*: *Lophiolepis* Cass., with 104 Eurasian and North African species, and *Epitrachys* (DC. ex Duby) K. Koch, comprising a single Mediterranean species. This taxonomic restructuring is supported phylogenetically [**1,2**], morphologically [**2,7**], and by genomic features [**7**]”.**

Of course, researchers are free to interpret results, which is why we do science. However, the authors are deliberately implying that our results support the polyphyly of *Cirsium*, which is speculative at best. Additionally, they avoid mentioning that we have a different proposal and interpretation and state that our results support the segregation of *Epitrachys*, even if that species was not included in our study (addressed in Section 2.4). In our papers, we always discuss all different proposals, including the one made by Del Guacchio et al. [2] from a holistic approach because, otherwise, we mislead readers. This seems to be the case in Bureš et al. [7]. It is our duty as scientists to use all the facts, not only those that benefit us. We find it unsettling that these authors suggest we are ignoring our plastid results, when indeed, we have discussed them extensively. Meanwhile, their papers focus solely on interpreting the results that support *Lophiolepis* as a different genus while overlooking other findings. This is what we find misleading. While we are on the topic, we would like to express our discontent with the use of the word “polemically” to refer to the discussion we wrote in Moreyra et al. [1]. We find it unprofessional and completely unnecessary.

In conclusion, while Del Guacchio et al. [2] have contributed to the discussion, their findings lack the necessary resolution and robustness to challenge the monophyly of *Cirsium* as supported by our nuclear phylogeny. Until more comprehensive data are available, we advocate for maintaining *Cirsium* as a monophyletic genus. If future research proves that *Cirsium* and *Lophiolepis* are not sister clades, clearly differentiated on the field, and are on an independent evolutionary history, we will be happy to accept the classification, as our objective is to shed some light on and solve the complex tangle that represents the evolutionary history of thistles.
